# Work outcomes in midlife women: the impact of menopause, work stress and working environment

**DOI:** 10.1186/s40695-018-0036-z

**Published:** 2018-04-09

**Authors:** Claire Hardy, Eleanor Thorne, Amanda Griffiths, Myra S. Hunter

**Affiliations:** 1 Department Psychology (at Guy’s), Institute of Psychiatry, Psychology and Neuroscience, Kings College London, 5th Floor Bermondsey Wing, Guy’s Campus, London, SE1 9RT UK; 2 Division of Psychiatry & Applied Psychology, School of Medicine, University of Nottingham, Nottingham, UK

**Keywords:** Menopausal status, Absenteeism, Job performance, Turnover intention, Intention to leave the labor force, Job stress, Working environment

## Abstract

**Background:**

There is growing research interest in the question of whether menopause impacts upon mid-aged women’s work outcomes, but the evidence to date is inconclusive. This paper examines whether: (i) menopausal status, and experience of hot flushes and night sweats (HFNS), and whether (ii) work stress and work environment, are associated with work outcomes (absenteeism, job performance, turnover intention, and intention to leave the labor force).

**Methods:**

An online survey (sociodemographic, menopause, health, well-being and aspects of work) was completed by 216 (pre-, peri- and postmenopausal) women aged 45–60 years.

**Results:**

Work outcomes were not associated with menopausal status but were significantly associated with job stress and aspects of the work environment, such as demand, control and support. HFNS presence, frequency and problem-rating were not significantly associated with work outcomes. HF problem rating at work was significantly associated with intention to leave the labor force, after controlling for age (F(2,101), 6.742, *p* = .002).

**Conclusions:**

The main predictors of work outcomes in this sample of mid-aged women were aspects of the working environment (particularly role clarity and work stress). Menopausal status was not associated with work outcomes but having problematic hot flushes at work was associated with intention to stop working. These results challenge assumptions about the menopause transition by providing evidence that the menopause does not impact on women’s self-reported work performance and absence. However, support for women with problematic HFNS at work may be beneficial, as might addressing working environment issues for mid-aged women.

## Background

The increasing age of the working population in most European countries means that more women will be working during their menopausal transition than ever before [1]. The menopause - the last menstrual cycle - generally occurs on average between the ages of 50 and 51 in western cultures [2]. The perimenopause or menopause transition is the time from the onset of menstrual cycle changes until one year after the final menstrual period [3]. Although highly variable between women, the menopausal transition can last on average two to four years, but can last up to ten years [4, 5].

It has been estimated that between 20 and 40% of menopausal women experience hot flushes and night sweats (HFNS), also referred to as vasomotor symptoms, which can impact negatively on quality of life, including personal and work life [6]. Women tend to report that these symptoms are more difficult to manage in the work place, due to embarrassment and concern about the reactions of others [7–9]. Menopausal status and HFNS are often kept hidden [10] and not disclosed to managers at work [11]; consequently menopause taboos are not challenged and women may not obtain practical support that could be helpful. This has led to various guidance and recommendations that menopause at work warrants attention and support for women. For example, the UK Faculty of Occupational Medicine of the Royal College of Physicians has published guidance on how employers can best support menopausal women in the workplace [12].

Recent research on this topic has focused on two main areas: (i) that menopause can have negative effects at work and (ii) that certain working environments negatively impact on experience of menopausal symptoms. A recent systematic review by Jack and colleagues [13] explored menopause at work and found a number of studies suggesting that women with problematic menopausal symptoms may experience impairments on a range of work outcomes. For example, in an Australian survey of approximately 1000 women aged 40–70 years, HFNS frequency was significantly associated with reduced job satisfaction, work engagement, organizational commitment, and a higher intention to quit their job [14]. In the US, a significant relationship was found between night sweat severity and impaired worker productivity on a large sample of over 3000 mid-aged women [15]. Overall, however, the systematic review concluded that evidence was still inconclusive in terms of the impact of menopause on work outcomes. It is important to note that almost without exception, evidence relating to performance at work is based on self-perceived measures.

Since the review’s publication, a further cross-sectional study of 1274 female workers aged 40–65 years in Australia found that having HFNS were associated with a greater likelihood of poor self-rated work ability [16]. In another recent study Hickey and colleagues [17] examined relationships between work outcomes and stage of menopause, in a study of over 1000 women in Australia. Self-reported work engagement, job satisfaction, organizational commitment, or work limitations did not differ with menopausal status. Postmenopausal women were less likely to report intention to leave their employing organization (turnover intention) than pre- or peri-menopausal women. However, the study did not control for the potential impact of age on these variables, which the authors noted may have been important to consider.

Finally, a recent report published in 2017 systematically reviewed the economic evidence of possible impact of menopause upon work outcomes, i.e. whether the menopause transition is a problem for UK working women and, in turn, workplaces and the wider labor market [18]. Both positive and negative effects were found for women transitioning whilst in employment, and some evidence suggested that menopausal women were unable to seek employment, were reducing their working hours, leaving or losing their job whilst in transitions, and identifying negative impact on their career. However, they, like Jack and colleagues [13], stated that evidence for the menopause having an economic impact remains inconclusive. It is also important to note that evidence relating to women’s performance is based on self-perceived performance not objective measures.

A key aspect of the workplace is job or work stress. Work-related stress is one of the main reasons reported for sickness absence in many developed countries; for example in the UK work stress has been the focus of much research over the last several decades [19]. Job stress is generally understood as a result of an employee’s cognitive appraisal that their working environment may be imposing greater demands (stressors) on them than they can cope with. The relationships between these cognitive appraisals and psychosocial environmental stressors has been largely influenced by Lazarus’s transactional model [20], which posits that stress results when person/environment transactions lead the individual to perceive a discrepancy between the demands of a situation and his/her resources or ability to cope with those demands. Stress has been examined in many populations and occupations, yet, mid-aged women have been largely overlooked.

Researchers have attempted to identify aspects of the work environment that might result in job stress and work outcomes. For example, early work was influenced by role stress theory [21] and the Person-Environment fit theory [22]. These theories proposed that the employee’s role was key and that if the employee did not fit the working environment appropriately then stress would occur. More recently this field has been more heavily influenced by Karasek’s jobs demands-control model [23] and the spin-off job demands-resources (JDR) model [24]. Within these theories, if an employee has insufficient control or resources or lack of support to be able to meet the demands of their job, then stress would occur. The JDR model [24] refers to control as a resource, specifically, but also suggests that other resources are available in the employee’s physical, psychological, social, or organizational domains.

In the context of female employees at midlife, there is a need for more research exploring job stress in menopausal women as well as the possible impact of menopausal status on work outcomes. This paper attempts to contribute by addressing the following research questions: (i) are menopausal status and the experience of menopausal symptoms – hot flushes and night sweats – associated with key work outcomes, including absenteeism, job performance, turnover intention (leaving the current employing organization), intention to leave the labor force, and (ii) what is the association of job stress and the working environment on these work outcomes?

## Method

An electronic survey was sent via email to female members of a trade union and professional association for family court and probation staff in England, Wales and Northern Ireland in June 2016. The workforce had undergone organizational change during the past three years and was considered a suitable population to explore job stress. Self-report data was collected on demographic and health-related questions, including age, ethnicity, educational level, general health, work-related variables included: employment status (full-time, part-time), working hours, flexible working, manual working, and managerial/supervisory responsibilities.

Job stress was measured using a single-item asking women how stressful they find their jobs (1 = not stressful to 4 = extremely stressful, [25]). The working environment was measured using the Health and Safety Executive’s Management Standards Indicator Tool (MSIT) which includes 35 items to measure six aspects of work which if badly managed are known to be associated with the experience of stress; demands, control, support (manager and peer), relationships, role and change [26]. Responses are given using a 5-point Likert-scale (1 = never to 5 = always) and were all shown to have acceptable levels of reliability (α = .68–.87).

Menopausal status was determined according to menstrual criteria: regular periods (regular for them), changes in menstrual periods but had menstruated in the last 6 months, or had not menstruated for a least 1 year. Participants were grouped as pre, peri-, and post-menopausal, respectively; perimenopause or menopause transition being the time from the onset of menstrual cycle changes until one year after the final menstrual period [3]. HFNS were assessed using the Hot Flush Rating Scale [7] including the presence of HFNS, HFNS frequency in the past week, HFNS problem rating (3 ten point scales items assessing interference, distress and problem ratings of HFNS α = .87), and an additional single item 10 point scale assessing HFNS problem rating specifically when at work.

Dependent variables were: number of days affected by work absence in the last 4 weeks (summed total number of full days, arriving to work late, and leaving early), a self-rated job performance item (a single item, 1 = poor-5 = excellent, [27, 28]), turnover intention was measured using an existing 4-item measure, with 5-point Likert scales, to assess the employee’s intention to leave the organization (α = 0.78) [29]), and intention to leave the work force was measured using a single-item (1 = no, 2 = sometimes, 3 = yes).

Univariate regression analyses were conducted to determine any significant associations between sociodemographic variables and the outcomes. Only age was significantly related to intention to leave the labor force only (*r* = .36, *p* = .000) and was controlled for in the main analyses. ANOVA was used to determine if there were menopausal group differences in the outcome variable absence; Kruskal-Wallis H Tests were used to determine if there were differences in perceived job performance and turnover intentions between the menopausal groups, and ANCOVAs were conducted to determine whether there were group differences in the outcome intention to stop working, controlling for age. Mann-Whitney U tests were used to determine whether there was an association between HFNS presence and the work outcomes. Bivariate linear regression analyses were conducted to determine whether experience of HFNS was associated with the outcome variables, and multiple regression analyses were used to determine the relative associations between significant univariate variables with the outcomes variable.

Ethical approval was given by King’s College London, Ethical Review Committee (reference number: HR-15/16–2492) and all participants gave their consent to participate and publish the results.

## Results

### Sample characteristics

Two hundred and sixteen women aged 40–65 years were included in this study. Table 1 shows the characteristics of the sample. Women were on average 53 years, white (88.7%), mostly educated to at least degree or professional qualification level (85.6%) and were generally healthy. Over half exercised a minimum of 2 times per week (55.1%). The sample’s mean BMI score (29.30) is considered at the upper end of being overweight.

**Table 1:** Sample characteristics

Characteristic		Mean (SD) or *N* (%)
Age (*n* = 216)	Mean (sd)	52.51 (5.75)
Ethnicity (*n* = 212)	White	188 (88.7%)
	Black	19 (9.0%)
	Asian	5 (2.4%)
Menopausal status (*n* = 216)	Pre (regular periods)	48 (22.2%)
	Peri (irregular periods for last 6 months)	42 (19.4%)
	Post (no period for 12 months)	126 (58.3%)
HFNS frequency in past week (*n* = 102)	Mean (sd)	19.45 (17.90)
		range: 0–89
HFNS problem rating (*n* = 104)	Mean (sd)	4.77 (2.11)
HFNS Problem rating at work (*n* = 104)	Mean (sd)	5.03 (2.71)
General health (n = 216)	Mean (sd)	2.81 (1.07)
BMI (*n* = 199)	Mean (sd)	29.30 (7.39)
Education level (*n* = 215)	‘O’ Grade/ ‘O’ Level/ Standard Grade	17 (7.9%)
	Higher/ ‘A’ Level/ National Grade	14 (6.5%)
	Degree or professional qualification	106 (49.3%)
	Post-graduate qualification	78 (36.3%)
Relationship status (*n* = 214)	Single/Divorced/Separated/Widowed	63 (29.4%)
	Married/With partner	151 (70.6%)
Work full-time (*n* = 212)		155 (73.1%)
Working hours (*n* = 211)	Mean (SD)	36.44 (9.68)
Flexible working (*n* = 212)		148 (69.8%)
Non-manual job (*n* = 215)		210 (97.7%)
Managerial/supervisory responsibilities (*n* = 212)		59 (27.8%)
Sector (*n* = 216)	Public	131 (60.6%)
	Private	85 (39.4%)
How stressful do you find your job? (*n* = 216)	Not stressful	5 (2.3%)
	Mildly stressful	35 (16.2%)
	Moderately stressful	95 (44%)
	Extremely stressful	81 (37.5%)

Fifty-eight per cent of the women were postmenopausal, with the remainder divided roughly equally between peri- or pre-menopausal. 62% were experiencing HFNS, on average, 19 times in the past week. Apart from one participant, none were taking HRT. A small proportion of women were taking non-prescribed medication for the menopause (7.7%) and fewer were taking prescribed medication (4.8%).

The majority of women worked full-time, for an average of 36 h per week in non-manual jobs (97.7%), within a public sector organization. Most of the women in the sample had degree level education and were employed in non-manual work. The majority (81.5%) were experiencing moderate to severe levels of job stress with only 2.3% reporting no job stress.

Women had been affected by absence, on average, for 4 days in the last 4 weeks. Specifically, they took an average of 2.37 (sd = 5.59) full days, arrived late to work 1.26 (sd = 3.56) times in the past 4 weeks, and left work early 1.13 (sd = 2.90) times in the past 4 weeks.

Self-rated work performance was generally perceived as high with three-quarters (74.9%) rating their performance as very good/excellent compared to others in a similar role. Approximately half of the women (52.3%) indicated that they have considered leaving the labor force altogether.

### Menopause and work outcomes

Table 2 shows the scores for the menopausal status groups on the key work outcomes.

**Table 2:** Total sample and pre, peri, and postmenopausal status groups on work outcomes

	Number	Pre-menopause *M *(SD)	Number	Peri-menopause *M *(SD)	Number	Post-menopause *M *(SD)	Number	Total *M *(SD)
Absence (Number of days affected by absence in last 4 weeks)	48	5.49 (6.59)	42	4.60 (6.13)	126	3.89 (6.20)	216	4.38 (6.28)
Job performance	47	3.00 (0.75)	39	2.87 (0.73)	125	2.86 (0.75)	211	2.89 (0.75)
Turnover intention	48	3.19 (0.99)	42	3.11 (0.99)	126	3.18 (1.04)	211	3.17 (1.02)
Intention to leave the labor force	48	0.48 (0.80)	42	0.64 (0.88)	126	1.07 (0.87)	216	0.84 (0.89)

No significant differences were found between menopausal status and any work outcome, i.e. number of days affected by absence (total number of days taken off, arrived late, left early), job performance, turnover intention. Intention to leave the labor force was significantly different between the menopausal groups, *H* = 19.300, *p* = .001, with post-menopausal women showing a significantly higher intention than pre- or peri-menopausal women. However, this difference became non-significant after controlling for age.

### Job stress and work environment

Relationships between job stress and work environment were considered as possible predictors of work outcomes. To improve normality of the job stress variable, responses were recoded to combine the lower two response options and create a 3-point scale (i.e. low, moderate, high stress) for use in the following analyses. Higher perceived job stress was significantly associated with lower self-rated job performance, F(1, 209) = 22.317, *p* = .0001, higher turnover intention, F(1, 214) = 37.016, p = .0001, and higher intention to leave the labor force controlling for age, F(2, 213) = 23.012, *p* = .0001. Job stress was not associated with number of days affected by absence.

Regarding the working environment, higher self-rated job performance was associated with lower demands, F(1, 209) = 11.59, *p* = .001, clearer job role, F(1, 209) = 30.53, *p* = .0001, and having more control at work, F(1, 209) = 7.771, *p* = .006.

Higher turnover intention was associated with higher demands at work, F(1, 214) = 18.575, *p* = .000, lower role clarity, F(1, 214) = 41.683, *p* = .0001, low control, F(1, 214) = 21.611, *p* = .000, better relationships, F(1, 214) = 4.019, *p* = .046, lower manager support, F(1, 214) = 23.99, *p* = .000, lower peer support, F(1, 214) = 10.155, *p* = .002, and poor change management, F(1, 214) = 24.467, *p* = .0001.

Higher intention to leave the labor force, controlling for age, was associated with higher demands, F(2, 213) = 20.307, *p* = .0001, poor role clarity, F(2, 213) = 5.826, *p* = .017, lower control, F(2, 213) = 16.199, *p* = .022, lower peer support, F(2, 213) = 19.766, *p* = .014, and poorer change management, F(2, 213) = 19.639, *p* = .015.

Absence (more days/part days off work) in the last four weeks were associated with lower perceived levels of control, F(1, 214) = 5.826, *p* = .017, and better relationships, F(1, 214) = 9.256, *p* = .003.

### The role of HFNS

A subsample (*n* = 168) of peri and post-menopausal women provided data relating to HFNS, and relationships between HFNS and work outcomes were examined. Neither the presence of vasomotor symptoms (HFNS) nor HFNS frequency or Problem-rating were associated with work outcomes. However, reporting more problematic hot flushes *at work* was significantly associated with intention to stop working, F(2, 101) = 6.742, *p* = .002. Specifically, higher problem ratings were associated with a greater intention to leave (*B* = .082).

### Relationships between age, job stress, working environment, HFNS, and work outcomes

Significant variables in univariate analyses were entered into stepwise linear regression analyses to determine the strongest predictors of each work outcome (see Table 3). Overall, the number of days affected by work absence was predicted by (better) relationships at work and (lower) control at work, but together these variables only accounted for a small (6%) amount of the variance. Job performance was best predicted by the working environment subscales, (higher) job role and (lower) job stress, which accounted for 16.1% of the variance. Intention to leave the employing organization was best predicted by (poorer) role clarity, (higher) job stress, and (poorer) managerial support, which accounted for 24.9% of the variance. Intention to stop working was best predicted by (older) age, (poorer) role clarity, and (higher) problematic hot flushes at work, which account for 22.5% of the variance.

**Table 3:** Predictors of work outcomes: step-wise linear regression analyses

Work outcome	Variable	Regression statistics
Number of days affected by absence (*n* = 216)	Relationships	B = 2.667, 95% CI = .746–4.587, *p* = .007
	Control	B = −1.498, 95% CI = −2.957--.040, *p* = .044
		R^2^ = .060
Job performance (*n* = 211)	Role	B = .324, 95% CI = .189–.459, p = .0001
	Job stress	B = .-.206, 95% CI = −.346--.066, *p* = .004
		R^2^ = .161
Turnover intention (*n* = 216)	Role	B = −.342, 95% CI = −.529--.156, *p* = .0001
	Job stress	B = .344, 95% CI = .164–.524, *p* = .0001
	Managerial support	B = −.184, 95% CI = −.317–.051, *p* = .0001
		R^2^ = 249
Intention to leave the labor force (*n* = 104)	Age	B = .072, 95% CI = .037–.107, *p* = .0001
	Role	B = −.264, 95% CI = −.475--.054, *p* = .014
	HF Problem rating at work	B = .065, 95% CI = .006–124, *p* = .031
		R^2^ = .225

## Discussion

This study contributes to the evidence regarding the potential impact of menopausal experience, and of work stress and work environment on work outcomes. The sample was highly educated and generally healthy, but reported moderate and severe levels of work stress and fairly high levels of work absence (2.37 full days absence in past 4 weeks), compared with a national average for annual sickness absence in the UK of 4.3 days [30]. There was not a particularly strong intention to leave their employing organization, but approximately half of the women had considered leaving the labor force altogether. Despite this, their subjective ratings of their own work performance were relatively high. The organization had recently undergone substantial change, which is known to be associated with a high report of stress. The results also suggest that performance remained high despite considerable work stress, absence and intention to leave the labor force. There is some evidence from qualitative data that women might work harder in order that their performance is not affected [11].

We found that there were no differences between pre, peri, and post-menopausal women with respect to work absence, job performance, turnover intention, and intention to leave the labor force. Neither were dimensions of HFNS reporting (prevalence, frequency and problem-rating) associated with work outcomes. However, having problematic hot flushes, specifically at work, was associated with a higher intention to leave the labor force. These results suggest that any impact of menopause or menopausal symptoms on work outcomes is likely to be minimal and quite specific. Interestingly, it is the problematic nature of HFNS, not their frequency that had an impact on the work outcomes studied here. This supports previous findings that it is how bothersome or problematic that HFNS are that is associated with quality of life, rather than their frequency [6].

Overall, our results support those of Hickey and colleagues [17] who found few differences between reproductive status on a range of work outcomes. Women rated their work performance positively in both studies. Hickey and colleagues found one significant association - that postmenopausal women had a lower intention to leave the labor force than pre- and peri-menopausal women. However, age was not controlled for in this study. In contrast we found that post-menopausal women reported a higher intention to leave the labor force than pre- or peri-menopausal women. However, this difference became non-significant after controlling for age.

The impact of work stress and the work environment was also examined and found to be significantly associated with key work outcomes. Successive Health and Safety Executive Labour Force Surveys on self-reported work-related illness have revealed that mid-life women (aged 45–54) are the group reporting most work-related stress [19]. We did not find an association between work stress and menopausal status. However, levels of work stress were relatively high across these stages. The results are similar to those reported by professionals in a similar occupational field (i.e. the police, [25]). In the UK, public service industries show the highest levels of stress and it is the main reason for absence from work [19].

Work stress and the working environment appeared to play a greater role in the work outcomes than menopausal status or experience. Specifically, job stress was associated with job performance, and turnover intention, although not significantly for days affected by absence. The working environment appeared to be more strongly associated with absence, especially having better relationships at work and less control over work. Job role clarity was also a key influence on these work outcomes, especially for intention to leave the employing organization and labor force. Managerial support was associated with turnover intention but none of the other outcomes. Intention to leave the labor force was additionally influenced by age and problematic hot flushes at work, which was the only outcome to be associated with the menopausal experience.

With regards to the menopause transition, there is guidance [1, 12] that encourages managers to be informed about the menopause and to foster a culture where women feel empowered to speak up about any difficulties. Increased flexibility, attention to workplace temperatures and access to information and advice are also recommended [11]. In a recent study of how mid-aged women wanted to be treated in the workplace [31] most women mentioned that employers/managers should not consider the menopause in an overly negative light, for example, as an ‘affliction’ or a ‘condition’ affecting all older female employees. They believed that employers/managers should be aware that the menopause is a normal process and one that is highly variable between women.

It is also important to mention other symptoms that may be associated with the menopause that we did not examine. These include tiredness, poor concentration and memory, and low confidence, and sleep disturbance [9, 11, 15]. Hickey et al. [17] found that sleep problems were most commonly reported by peri-menopausal women, while for postmenopausal women it was joint and muscular discomfort. Only hot flushes and vaginal dryness were significantly more frequent in peri- and post, compared to premenopausal women. Whitely and colleagues [9] concluded, from a study examining the effects of menopausal symptoms on work impairment, that whilst women with menopausal symptoms reported significantly higher work impairment, there was no specific symptom that significantly predicted work productivity losses.

In this context and in the light of the current findings and those of Hickey et al. [17], we suggest that specific symptoms or physical changes, such as HFNS, are considered since menopausal status does not appear to be associated with most work outcomes. This is likely to reduce general stereotyping of ‘menopausal women’ and address women’s concerns about being perceived as ‘not good at their jobs’ because they are going through the menopause. In addition, work outcomes, such as performance and work intentions, appear to differ markedly in their relationship to HFNS, so future studies might usefully include a broad range of work outcomes. It is also important to report positive findings, for example in the current study work performance was highly rated in this sample of working mid-aged women.

Attempts to retain women in the labor force might focus on providing support to those women who are having problematic HFNS specifically at work, as well as modifying aspects of the work environment that can exacerbate experience and women’s ability to cope with symptoms [1, 11, 12]. Information and advice on managing symptoms using a cognitive behavioural approach is available [32, 33], including a self-help approach for working women with problematic symptoms [28], and may be offered as needed. The results also suggest that steps need to be taken to help employees to have clarity of roles, feel supported, and have more control over their work.

Future research might investigate changes at work and their impact on menopause experience: for example, providing information and training about the menopause to all staff. Such enquiries might compare different delivery methods (face to face, paper or online). Research might explore attempts to improve workplace culture regarding health-related issues for women. The effectiveness of risk assessment and risk management initiatives could be explored, where key factors affecting women are identified and interventions designed to reduce them.

Some limitations should also be noted. The overall sample size was relatively small and also derived from one job sector (i.e. the probation service) that had undergone organizational change in the past 3 years. This may influence the generalizability of the findings, and further research exploring a range of different job sectors is recommended.

The women appeared to be experiencing relatively high levels of stress, but we did not explore non-work sources of stress, which are commonly reported during midlife, such as caring roles and family responsibilities [34].

## Conclusion

This study presents evidence that menopausal status does not appear to be associated with work outcomes (absence, performance, turnover intention and intention to leave the labor force) and most women maintained high levels of self-rated performance at work despite menopause and high levels of work stress. The results therefore challenge the assumptions that the menopause has a negative impact on work performance and levels of absence from work. The findings suggest implications for possible changes to workplace practices and policy that may benefit those mid-aged women experiencing difficulties during mid-life and/or the menopause. In particular, providing support for women with problematic HFNS at work, as well as addressing working environment issues. Investigations examining the impact of such workplace changes and tailored interventions are needed.
